# Waves of hurt faced by families due to a hospitalised relative with COVID-19 in the Cape Flats

**DOI:** 10.4102/hsag.v30i0.2754

**Published:** 2025-03-25

**Authors:** Mondli Chiya, Karien Jooste, Amy Williams

**Affiliations:** 1 Department of Nursing Science, Faculty of Health and Wellness Sciences, Cape Peninsula University of Technology, Bellville, South Africa

**Keywords:** COVID-19, family, experiences, comfort, support, hurt, community, unforeseen hospitalisation

## Abstract

**Background:**

Family members value each other, particularly during troubled times, and the consequences of a sudden disruption to their family environment could imply difficult adjustments, affecting their well-being. They did not expect that a family member would get severely ill due to coronavirus disease 2019 (COVID-19). It was unclear what the experiences of family members were on the sudden hospitalisation of a close relative diagnosed with COVID-19.

**Aim:**

To gain insight into the experiences of family members with a close relative unexpectedly hospitalised with COVID-19.

**Setting:**

Two health clinics in Cape Town, were identified that family members visited after the hospitalisation of a close relative with COVID-19.

**Methods:**

A descriptive phenomenological design was followed including 11 participants, heterogeneously purposefully sampled. Individual interviews lasted 30 min– 45 min, posing semi-structured questions - probing led to thematic saturation. Data coding resulted in four themes with 11 categories.

**Results:**

Nursing communication with communities during COVID-19 could identify the presence of emotionally wounded states of family members. Nurses should be competent in techniques to relieve the fear of families around their isolated hospitalised relatives. Further research can explore family-orientated interventions needed to deal with fear of losing a hospitalised relative being critically ill.

**Conclusion:**

The need of families for ‘nearness’ to a close relative needed to be understood - it meant more than physical proximity.

**Contribution:**

Mindful nurses should act as comforters to families to relieve fear around possible changes in an unknown future due to a critically ill family member.

## Introduction

### Background

Global epidemic outbreaks have negative effects on individuals and society (Duan et al. 2020) and, on the other hand, bring a greater awareness of the closeness of family members during these stressful situations. Families differ in many facets, but what is similar is how family members value each other in one way or another, particularly during challenges and especially when a family member is admitted to a hospital (Bartoli et al. 2021:927). Dermott and Fowler (2020:6) conceptualise the word ‘family’ and argue that a modern way of understanding a family is to look at it as where two or more people share the same goals and values while having a long-term commitment to one another and living together. How each family experiences the repercussions of coronavirus disease 2019 (COVID-19) is impacted by the presence of vulnerable parents, children, the socio-economic status and conditions, and the psychological makeup of people (Jackson et al. 2020; Pragholapati 2020:3).

When a family relative becomes sick, it affects the well-being of other family members and causes changes in the lives of all of them. In this study, a close relative referred to a brother, sister, husband, wife or person staying as the family in the same household. Evidence has shown that the reaction of family members to a sudden occurrence such as a loss of a family member involves stress, confusion and uncertainty, a search for information, and attempts to fulfil the perceived needs of the patient and themselves (Terzi et al. 2022:22). However, research suggests that family members do not always perceive that they are included in the treatment of their relatives. Furthermore, they believe nurses do not adequately address their needs (Picacrdi, Miniotti & Leombruni 2021:162).

The COVID-19 pandemic posed an unprecedented challenge to science and society. It demanded rapid and diverse responses from health systems that needed to reorganise all their components (Medina et al. 2020:1). The COVID-19 emerged as an outbreak of respiratory sickness (Cheng et al. 2020:748).

In March 2020, the National Institute for Communicable Diseases confirmed that a suspected case of COVID-19 had tested positive in South Africa. At that stage, South Africa already showed that there were 1326 positive cases in the country, with 324 reported cases in the Western Cape province and a total of three deaths nationwide (Gibson & Rush 2020:3). The COVID-19 epidemic posed a remarkable threat to the health of the community (Pan & Zhang 2020:1; Zhao et al. 2022:1), but this health crisis did not affect everyone in the same way (Jackson et al. 2020). During this time, people were not allowed to leave their residences other than for medical attention and essential goods and services (Gittings et al. 2021:948).

## Literature

A global outbreak of respiratory sickness, later shown to be caused by a novel coronavirus, and officially termed COVID-19, was first reported in Wuhan, a city in Hubei province, the People’s Republic of China, on 31 December 2019. In response to the COVID-19 pandemic, a nationwide lockdown was announced for 21 days on 26 March 2020 (Gibson & Rush 2020).

There were various adverse economic and social disruptions during COVID-19. There was, for example, strictly regulated human movement and transport to monitor and combat the epidemic’s growth. This placed pressure on individuals to conduct their daily work and social responsibilities (Mofijur et al. 2021). Duan et al. (2020) argue that the adverse effects of the COVID-19 pandemic were a shock of global proportions, impacting families when their members contracted COVID-19. Coronavirus disease 2019 caused an increase in stress when a relative had a member admitted, or there was a sudden admission of a family member to the hospital, as well as an economic burden when the breadwinner of the family contracted the disease (Jackson et al. 2020; Pragholapati 2020:3). Family members of ill patients with COVID-19 experienced a psychological toll on them due to feeling stressed and having anxiety and fear. They worried about their financial status and the lack of sufficient family support as the relative mainly was the breadwinner (Asadi & Salmani 2024).

Nurses need to be sensitive to family members’ emotions, identify problems and help them cope with the situation (lack of proximity) and feeling isolated from their loved ones. Apostol-Nicodemus et al. (2022:9) argue that a family member’s anxiety stems from the inability to feel connected to the patient and to be informed about the delivery of care. Regular, updated information on the patient’s condition will enable families to understand and cope with the situation (Apostol-Nicodemus et al. 2022:9; Zainah et al. 2016).

The COVID-19 pandemic required a focus on community engagement as a process of working collaboratively with the concerns of families with a member in a hospital with COVID-19 to incorporate their interests in decision-making processes (Turin et al. 2020:7). Interacting with a vulnerable population such as traumatised family members and the role of the nurse in engaging with them can address grassroots concerns in the process of making informed decisions (Turin et al. 2020:9). Research suggests that family members do not always perceive that they are included in the treatment of their relatives; therefore, they believe that their human needs are not adequately addressed by nurses (Kynoch, Cabilan & McArdle 2016:24; Picacrdi et al. 2021:162). The research question was: *How was it for family members who experienced the unexpected hospitalisation of a close relative with COVID-19?*

## Theoretical assumptions

The Critical Care Family Needs Inventory (CCFNI), developed by Molten in 1979, and revised by Leske in 1991, confirms the essential basic needs of family members as having information, assurance, support, closeness or proximity, and comfort (Chhetri & Thulung 2018:10). This family-centred care (FCC) framework indicates the predicament of family members with a hospitalised relative. It has generated much interest in FCC in the community. The framework further outlines that nursing practitioners work from a guiding assumption that ‘health and illness is a family affair’ and that patients and their families are inextricably connected. In this study, it was assumed that:

- The basic needs of family members with a close relative who has been hospitalised with COVID-19 should be assessed and supported adequately by nursing staff.
- Complete and honest information should regularly be provided to the family members on the patient’s (relative) health condition within a nursing professional-ethical framework.
- Closeness to the patients included more than standing at the bedside.
- Comfort means viewing a family member of the patient holistically to ease the impact of the situation around the patient with COVID-19.

The purpose of the study was to understand the lived experiences of family members when a close relative with COVID-19 was admitted to a hospital.

### Design

Husserl’s (1859–1938) descriptive phenomenology design was followed to understand and capture the essence of the lived experiences around the phenomenon (Mbaka & Isiramen 2021:29) rather than an interpretive phenomenology design in which the voice, situation and context of the phenomenon come to the fore (Öhlén & Friberg 2023).

### Setting

A township in the Western Cape Province of South Africa, on the Cape Flats in the City of Cape Town Metropolitan Municipality, was the study location where data were selected between September and October 2021. Two of the 11 clinics situated throughout this township, viewed as providing the most comprehensive primary health care services, were included in the research. Services offered at these two clinics included child health, family planning, tuberculosis treatment, HIV testing, Pap smears and treatment and diagnosis of sexually transmitted infections. As many as 391 749 people were living in this township according to the 2011 census.

### Study population and sampling

The accessible population for the study was family members who visited a local community health clinic after a close relative had been hospitalised with COVID-19. Heterogeneous purposive sampling was the best approach to follow in the big township, as it provided maximum variation sampling to include a diverse range of cases relevant to the research problem. This sampling technique provided as much insight as possible into the event or phenomenon under examination (Rahman 2023:49). Data and meaning (essence of the lived experienced saturation) were obtained by interviewing participants until no added codes or meanings of the topic emerged (Hennink & Kaiser 2022:2), bringing the total to 11 participants. Inclusion criteria comprised family members (older than 18 years) with a close relative. The study excluded participants who previously had COVID-19, as their own personal experiences could have been difficult to bracket in answering the research question that focused on their relative.

## Research methods and design

Phenomenological interviews were the method chosen to gain insight into the nature of the experiences of family members with a close relative unexpectedly hospitalised with COVID-19. Individual interviews were conducted and chosen instead of focus groups, as they provided comprehensive, contextualised data from the research participants regarding their specific experiences (Tanwir, Moideen & Habib 2021:11). The researcher thought about his own experiences with COVID-19 in his workplace and wrote reflected notes, thereby bracketing from the lived experiences shared by the participants, whom he did not know.

### Data gathering

Data were collected between September 2021 and October 2021. The first step of data gathering was the preparation of the field, during which the researcher visited the nurse managers of the clinics and professional nurses to explain the research purpose, obtain permission to include the clinics in the study and explain the inclusion and exclusion criteria that would be used for the selection of study participants. A request to use a quiet and private room in the clinics, away from the noise, was granted. Permission was approved to place a notice at the door entrance of the clinics providing information on the research study and purpose, with a telephone number for those family members who were interested in participating to contact the researcher. The professional nurses of the two health clinics were requested to assist with recruiting eligible participants after they had consultations with the patients and referring them to the researcher. A convenient date and time to meet in the pre-arranged private room were arranged. A pilot interview was conducted with an eligible participant referred to the researcher by a professional nurse at one of the clinics. The pilot interview was conducted similarly to the interviews in the main study. After the pilot interview, no changes were made to the questions, and the data obtained was included in the analysis of the main research. An interview schedule was used containing two semi-structured questions posed during in-depth individual phenomenological interviews, conducted in the language of preference, English or isiXhosa, both of which the researcher spoke well and could later directly translate. All participants gave written consent to being interviewed and their voice were digitally recorded. Participants were encouraged to speak freely with the researcher without the risk of being overheard or having any disturbances. Field notes were taken to write down the physical and non-verbal reactions of participants observed during the discussion on their experiences of their hospitalised relative. Probing questions were asked where clarity was needed, and the interviews lasted 30–45 min.

### Data analysis

The Colaizzi coding method (1978) offered a way to analyse data and develop trustworthy findings, and data were interpreted and coded along with the field notes. The interviews carried out in the local language of isiXhosa were translated into English by the researcher, and the independent coder also spoke both languages to ensure the integrity and accuracy of the data transcriptions. The researcher listened to and checked the voice recordings against the transcribed text. The data were carefully listened to and transcribed in the exact words used initially, and field notes were carefully read several times for accuracy (Lee et al. 2022:865). To determine which information should be included in a transcription, the unit of analysis was established before beginning data transcription. Concurrent coding of field notes and interview data were done. The steps in the Colaizzi’s descriptive phenomenological approach were followed, namely familiarisation with the data, identifying significant statements, formulating meanings, clustering themes, developing an exhaustive description, producing the fundamental structure and seeking verification of the fundamental structure. An independent coder and the researcher held a consensus meeting and agreed on the themes and categories that emerged from the data.

### Ethical considerations

An application for full ethical approval was made at Cape Peninsula University of Technology to the Higher Degree and Ethics Committee of the Faculty of Health and Wellness Sciences (Ethical clearance no.:CPUT/HWS-REC 2021/ H13), and ethics consent was received on 20 April 2021.

The nurse managers of each clinic were contacted to request permission to enter the sites. Permission was granted based on the submission of the proposal and ethics clearance from an approved national ethics committee. The information sheet with the research’s purpose, benefits and process was explained to the participants. Written informed consent was obtained from all individual participants involved in the study.

The anonymity of the participants was ensured by not mentioning their names on the digital recording, field notes and transcripts (transcripts were numbered). The researcher requested the participants to keep the information shared during the interview confidential, and only the researcher and independent coder had access to an online Google Drive file with data, which was password protected. Field notes were kept in the researcher’s locked safe. Data will be kept for 5 years until the report has been published, and then the electronic files will be deleted and hard copies shredded. Participants were free to withdraw from the interview without facing any repercussions. A psychologist was arranged to be in the clinic when interviews were conducted due to the sensitive nature of the research. Although it was unnecessary, if a participant became very emotional during the interview, it would have been discontinued.

## Results

Participants comprised two males and nine females aged 18 years–25 years, including the pilot interview participant. The mean age of the participants was 38 years, and their ages ranged from 21 years to 55 years. All participants had had a family member admitted to a healthcare facility with COVID-19. However, only three participants had relatives who survived after being admitted. Nine of the 11 participants had children. Seven were working, two participants were attending school or a college, one was self-employed, and another was unemployed.

The study aimed to gain insight into the nature of the experiences of family members whose close relative was unexpectedly hospitalised with COVID-19. Four themes emerged from the data analysis. The underlying story was that family members experienced curiosity and anxiousness in an indefinite time span, reality with insufficient information, and being far removed from their family relatives.

This article focuses on one of the themes, ‘Comfort should be given to the family members through comprehensive support to diminish “the waves” of hurt created by the unforeseen impact of COVID-19’ (Box 1).

BOX 1 Themes.

**Table:** 

Theme 1: Authentic information should regularly be made available to relatives to establish a trusting relationship with staff
Theme 2: Essential needs of family members must be identified to assist in relieving their pain
Theme 3: The need of families for ‘nearness’ should be understood, and means more than physical proximity to a close relative
Theme 4: Comfort should be given to the family members through comprehensive support to diminish ‘the waves’ of hurt created by the unforeseen impact of COVID-19

COVID-19, coronavirus disease 2019.

The highlighted theme (Box 1) gave insight into the comfort that should be given to the family members through comprehensively supporting them to diminish ‘the waves’ of hurt created by the unforeseen impact of COVID-19. Comfort is a multidimensional structure within the context of four experiences–physical, environmental, psychospiritual, and socio-cultural – and is defined as a condition for meeting basic human needs for relief, ease, and transcendence of suffering (Terzi et al. 2022:7).

**FIGURE 1:**
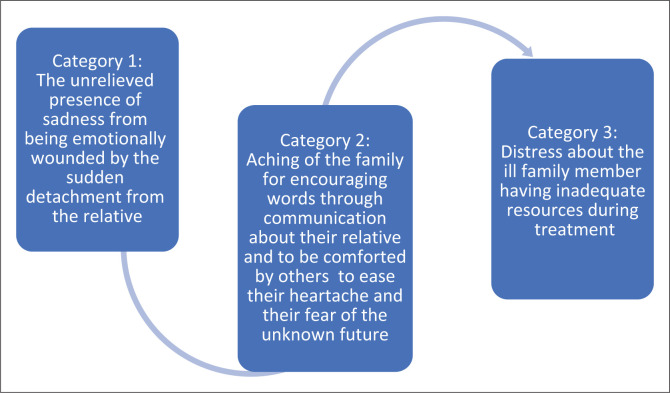
Waves of hurt.

Three categories emerged from the theme of providing comfort to the family members (Figure 1).

### Category 1: The unrelieved presence of sadness from being emotionally wounded by the sudden detachment from the relative

It was observed that the uncertainty caused by not knowing how a hospitalised loved one’s health condition was, or even worse, the absence of a final goodbye and sudden death of a family relative, created emotional wounds, from the painful experience, that seemed to have a profoundly impactful, that could shape their lives.

It was observed (from the facial expression of a participant) that she was in a state of mental distress and anguished as she shared her experience, saying:

‘It has brought sadness into our lives.’ (Participant 5, Female, 38 years old)

Family members reported suffering immensely after separation from their ill relatives and experienced these unfortunate happenings very intensively.

Circumstances provoked a complete sense of disbelief and alienation of the reality in the mourners, who had trouble coming to terms with the sad news:

‘Yoh [*exclamation of surprise*], it was sad … I am still very hurt even now [*emotional*] … I’m still devastated every time I think about it.’ (Participant 1, Female, 24 years old)

A sister of a deceased mentioned that it was the most tragic and dreadful experience in her life, which caused extreme suffering and distress. She was going through agony as she had recently lost three other family members as well:

‘My mom, my dad and my brother. They all died of COVID-19 … Yoh [*exclamation of surprise*], it was the worst time in my life.’ (Participant 9, Female, 48 years old)

The findings on participants being separated from their relatives brought emotional distress and grief that was difficult to cope with and come to terms with. During COVID-19, families had difficulties in coming to terms with and processing the losses due to the limitations of home confinement that in and of itself was an abnormal situation and prevented them from connecting with the reality of the worsening health and subsequent death of their family member (Hernández-Fernández & Meneses-Falcón 2022:1229).

### Category 2: Aching of the family members for encouraging words through communication about their relative and to be comforted by others to ease their heartache and fear of the unknown future

Family members felt that by not being present at the bedside with the hospitalised relative, they were missing critical information and conversation concerning their loved ones. This affected them negatively, as they were unable to communicate with their relatives and, therefore, had a need to be comforted by others to help ease their grief and emotional pain. Two sub-categories emerged from Category 2.

#### Sub-category 2.1: The need to be comforted by others and receive updated and adequate information about the relative to ease the pain

The support of family and friends appeared extremely important to those affected, as their relatives were hospitalised with COVID-19. Reassurance to family members was required through informed communication. Nurses seemed to be an essential communication resource for family members. A sister of a late hospitalised relative expected the nurses to provide personal orientated comfort to them by checking up on her and her family’s welfare after their relative passed away from COVID-19:

‘During that time, I needed someone I could talk to. I thought they [*nurses*] would check up on us and see how we are doing or do a home visit and test the rest of the family members for COVID-19 since one of my family members died of COVID-19.’ (Participant 5, Female, 38 years old)

It seemed that the family’s hurt was of such a nature that a personal home visit was expected to express condolences.

The need of family members to be communicated with and supported by others at the time that their relatives were admitted to a healthcare facility was mentioned:

‘Talking to friends and family encouraging me to stay positive.’ (Participant 3, Female, 55 years old)

‘I needed emotional support, like people calling and checking on me.’ (Participant 11, Male, 38 years old)

An urgent call was made for more timely and regular healthcare updates about relatives, and heartache was perceived in their quest for exceptions to be made to allow for face-to-face interactions despite visitation restrictions. It was said:

‘I wish that someone could just call me and let me know everything was going to be all right. I was not even allowed to see her in ICU because she was very sick, and people were not allowed to visit their family member in hospital.’ (Participant 11, Male, 38 years old)

The hurt of the family was demonstrated by them nearly ‘begging’ to be informed about the circumstances of their relative that they did not understand and found heartbreaking.

The need of family members to be reassured and nurtured through communication by others at the time of their relatives being admitted to a healthcare facility was essential:

‘Talking to friends and family encouraging me to stay positive.’ (Participant 3, Female, 55 years old)

A participant felt hurt, as she expressed how she was deserted and felt alone, especially after realising that her family would never see their loved one ever again:

‘I think the support I needed then was more after he died when than when he was still hospitalised. I just needed to be around people because when I was alone, I was thinking too much and stressing a lot. I think nurses can help us by telling us about the condition of the patient whenever we call to ask.’ (Participant 8, Male, 42 years old)

The support of family and friends appeared extremely important to those affected during their relatives being hospitalised with COVID-19.

#### Sub-category 2.2: Support to family members regarding their emotional, social, and financial challenges

Nurses should focus on their professional responsibilities in providing for the psychosocial well-being of the family members. One participant described how the counselling sessions by nurses helped her to cope and relieved her stress related to grief after the loss of a family member:

‘I did get support at clinic from one of the professional nurses, and as well as my family members were with me throughout the process. I also got support from my other sister, who’s a nurse also working in Joburg. She would call me to ask how I am doing. I’ve also went for a counselling session for six weeks at the clinic.’ (Participant 7, Female, 52 years old)

Some participants needed family support. The kind of support that the participants in this study seemed most in need of, and which they valued most, was emotional support:

‘The only thing I needed that that time was to see my sister and talk to her, but I couldn’t because we were not allowed to visit. I think nurses can help support families during COVID-19 when their close relatives are admitted by also assisting those patients who are unable to answer video calls so that they can see because family members could not see, touch, or hold their loved ones.’ (Participant 6, Female, 48 years old)

A daughter of the relative mentioned that she received psychological support, which was beneficial, and therefore suggested that relatives who had a family member admitted with or who died of COVID-19 should seek professional assistance and guidance in resolving personal or psychological problems:

‘I went for counselling, which really, which helped me a lot. I would recommend that to everyone who lost family member due to COVID-19 because it’s an instant thing, it’s a lot to deal with, you don’t get closure, it’s a lot to digest at once. So, it helps to speak with someone, a psychologist; it helps to deal with the trauma.’ (Participant 4, Female, 28 years old)

A family member mentioned that he needed to talk with others as his life was suddenly changing and that he was used to depending on the admitted relative:

‘Well, I needed financial support and emotional support. There was no one to talk to, and I also had to take care of my siblings.’ (Participant 11, Male, 38 years old)

### Category 3: Distress about the ill family member having inadequate resources during treatment

Health professionals were in extraordinary situations where there was a high demand for care, often accompanied by a shortage or scarcity of resources, resulting in the health system operating at saturation point (Delgado 2022:44).

A participant described how the shortage of equipment (oxygen) negatively influenced the care that her family member received. She mentioned that patients had to be prioritised according to their oxygen saturation levels:

‘At that time, the hospitals were full, and there was not enough oxygen therapy at the facility she was admitted. At the hospital she was admitted, they used to share oxygen when one patient’s oxygen saturation increased to near to normal, then they would take them off oxygen and put it on someone who had low oxygen saturation.’ (Participant 9, Female, 48 years old)

The participant further mentioned that the situation was out of the nurses’ control, as there were no beds available to admit patients, apart from the shortage of oxygen therapy:

‘I could see that situation was beyond their control; it was bad at the facility where my parents were admitted. Nurses tried their utmost best, but they could only do as much; sometimes, there were no beds for to admit patients or patients sharing oxygen. Basically, everyone was burdened.’ (Participant 9, Female, 48 years old)

The availability of healthcare providers and support staff played a significant role in the patient’s clinical management. The findings indicated that inadequate and inequitable staff distribution was a healthcare system factor contributing to COVID-19 mortality.

‘It would be nicer to get someone from the hospital to keep the family updated, maybe two-hourly, just to let the family members know because it’s not easy to have someone and not knowing anything about them. You keep calling their phones, and they not answering and the last time you spoke to them. they were out of breath.’ (Participant 2, Female, 28 years old)

Evidence alludes to the importance of having someone from the hospital update relatives by daily telephone contact regarding their family members. With this way of communication, family members can feel part of the situation, be recognised as family members and be taken seriously in their quest for reassurance of the well-being of their hospitalised relative (Klop et al. 2021:5).

### Trustworthiness

To ensure the quality of this study, the researcher relied on the five criteria offered by Lincoln and Guba (1985) to establish trustworthiness by ensuring credibility, which corresponds to its validity, dependability, confirmability (reliability) and transferability of the findings. Credibility was ensured through triangulation, involving using different data collection methods (interviews, field notes) to ensure consistency of the findings. Dependability was ensured by using an independent coder who co-analysed the data and reached a consensus with the researcher. This study provided a thick description of the methodology that was followed, and the recommendations formulated that made it potentially useful for evaluating the relevance and applicability of the study to other contexts, situations and populations. The researcher conducted critical reflexivity after interviews, which enabled him to avoid his own opinions being included in the data analysis (Amin et al. 2020:1478). The criterion of fairness in authenticity was followed in that the researcher had a prolonged engagement with participants in the clinics and ensured that he understood the participants’ experiences as they meant it. The researcher captured all the participants’ experiences, and he could convey the feeling and tone of their lived experiences (Peck & Mummery 2023:6).

## Discussion

Coronavirus disease 2019 detrimentally impacted families and acquaintances globally, directly and indirectly (Anthony et al. 2024). For different reasons, the separation of the family from their relative during COVID-19 was particularly problematic and disruptive in terms of the coping and pain processes of families (Bartoli et al. 2021:926). The unrelieved presence of sadness of the participants indicated that they were wounded emotionally by the suddenness of the hospitalisation or death of a close relative. Coronavirus disease 2019 posed a threat to one’s very existence (or that of relatives), a kind of threat that, according to much research, is deeply interrelated with a lack of cognitive and emotional control (Wnuk, Oleksy & Maison 2020:3). Some family members suffered from mental distress after separation from their ill relatives or the deceased. The absence of being able to have spoken to or said farewell to a loved one caused severe grief. Grieving is associated with disbelief, denial, a lack of acceptance, and coming to terms with a loss (Hernández-Fernández & Meneses-Falcón 2022:1230).

Encouraging communication from others to the families about their loved ones eased the hurt and fear of the unknown future. During an unfortunate period of being separated from a loved one, family members needed reassurance that could relieve the hurt and anxiety brought about by future uncertainties and the sudden hard-hitting transformation in the lives of their families. Visiting restrictions limited the presence of family members in the hospitals to provide emotional support for relatives, restricted FCC, and reduced opportunities for formal and informal communication with the healthcare team (Rose et al. 2022:1157). There was thus a need to know that the relative’s loved one had not been abandoned and was being taken care of.

Some families lost more than one relative, which was difficult, painful and challenging to come to terms with. This came along with the abnormal situation of being confined to their homes. Coronavirus disease 2019 in itself was an abnormal situation and prevented family members from connecting with the reality of the worsening health and subsequent death of their close relative (Hernández-Fernández & Meneses-Falcón 2022:1229). Coronavirus disease 2019 posed a threat to the very existence of relatives and led to deep emotional hurt. The separation of the family from their relative is particularly problematic and disruptive in terms of the coping and pain processes of families for varied reasons (Bartoli et al. 2021:926).

Reassurance by nurses through open communication and being with the family for support under the COVID-19 circumstances eased the pain and provided opportunities for expressing their fears of financial burdens and feeling alone. Clear and complete communication by healthcare providers could lessen the bereaved relatives’ hurt. Families especially appreciate initiative-taking and regular, accurate and sensitive communication during end-of-life care (Selman, Sowden & Borgstrom 2021). The loss or separation from a loved one can leave a sense of a void due to changes that happen in the daily routine of the family, and it may be that the relative was an important resource on which the family members relied (Cipolletta, Entilli & Filisetti 2022:988).

Family-centred care, including communication, collaboration and (bereavement) support, should be a core element of a high or intensive care unit when the patient’s situation is critical (Klop et al. 2021:2) to lessen the lasting emotional effect on families after the trauma and hurt. Previous studies of the COVID-19 pandemic have revealed that the psychological effects of infectious disease outbreaks can last long after the event, negatively impacting psychological well-being and causing post-traumatic stress disorder, depression, and stress among healthcare workers (Alnazly & Hjazeen 2021:263).

Family members needed support at the time that their relatives were admitted to the hospital or when they heard about the hospitalisation of the relatives to the facility. A common impulse among those experiencing grief is to seek comfort in the arms of family, friends and community (Selman et al. 2020:84). The rapid deterioration witnessed in many patients with COVID-19 who were admitted to hospital caused a high burden of stress, anxiety and feelings of loneliness among relatives (Klop et al. 2021:2). Therefore, the support of relatives of patients with COVID-19 admitted to a healthcare facility appeared to be extremely important.

Families viewed nurses as the liaison and essential resource when a relative’s health deteriorated. Support was needed, among other things, for information about the relative’s condition, comfort by knowing the patient’s status, and not feeling lonely during the hurt. Communication in end-of-life care is critical, and health professionals should be educated to provide this type of support, which is pivotal for both the patients preparing for their and their family members’ departure from life (Coppola et al. 2021).

The families’ need for reassurance about the health status of their ill relatives required professional support from nurses. Nurses have professional and moral responsibilities in providing for the psychosocial well-being of the family. Nurses’ understanding of providing holistic care requires them to intervene by evaluating family members physically and psychologically and determining risky conditions (Terzi et al. 2022:2).

It was mentioned that counselling was needed after the loss of a close family relative. In a culture where extended family ties are highly valued, South African families may also rely on other family members for counselling support rather than seeking this from health professionals (Redley et al. 2019:2819). While family, friends, and existing networks are the foundation of bereavement support – and for many people, the only support needed – formal bereavement services could also play a central role in supporting individuals and families (Selman et al. 2020:84).

Health professionals were in extraordinary situations where there was a high demand for staff to provide care and resources for the health system to operate. The worsening COVID-19 pandemic in South Africa posed multiple challenges for clinical decision-making in the context of already scarce resources (Naidoo & Naidoo 2021:1). Participants complained about several limitations in service delivery of the hospital and mentioned the poor nursing care provided by staff and the lack of beds for patients to be admitted to the intensive care unit. A study by Delgado (2022:44) confirms that the availability of healthcare providers and support staff played a significant role in the COVID-19 patients’ clinical management. Hai et al. (2024) add that optimised resource allocation for appropriate treatment is crucial in managing COVID-19 cases.

The strengths of this study were that an appropriate design was implemented to describe participants’ lived experiences. Phenomenological individual interviews allowed for the development of a trusting relationship with the researcher, who eased their ability to share their realities around the hospitalised relative. A limitation could be that purposive sampling and data saturation led to interviews with 11 family members, who could be viewed as a small sample, although a qualitative study is contextual. The study context implied that the findings could not be generalised; however, through transferability, it would be possible for other similar settings to evaluate the appropriateness of the study findings for their contexts.

### Reminder

The findings indicated that family members who experienced curiosity and anxiousness should be shown empathy, as they are indefinitely awaiting the health outcome of their close relatives with anticipation. The emotions of family members fluctuate between disbelief and mourning, and, as ‘waves of hurt’ are a reality for family members, sufficient information should be provided about the recent health status of the patient. This is crucial, as the family members experience that they are, in reality, far removed from their family relatives.

Comfort should be given to family members who have been emotionally traumatised by comprehensively supporting them to diminish ‘the waves of hurt’ created by the unforeseen impact of COVID-19. This could be done through several approaches. Firstly, after a family member has been assessed and found to have unrelieved sadness related to being emotionally wounded by the suddenness of loss, referral to a medical practitioner is a priority. Secondly, nurses should explore more creative means of communication between the nurse-patient-family triangle. This could be done in diverse ways, such as by sending messages through an information station monitored by a professional nurse or trained staff member of a ward. Thirdly, specific communication strategies could increase family satisfaction. This could include empathic statements to assure family members of non-abandonment, arranged motivational discussions to establish feelings of comfort around how the treatment is delivered, and provision of written information on the status of recovery of a patient (Selman et al. 2020:82). Reassurance strategies to relieve fear in the lives of families can be through showing them video clips of similar situations in a secluded area near the ward where the patient is.

Patient care should be delivered within a professional-ethical-legal framework. Good record-keeping, an integral part of competent professional practice, should be kept for possible legal cases after a patient has passed away. Nurses have a legal obligation to comply with the compulsory pandemic control measures of the employer and national government. Nurses should offer the best possible family-centred safe care to patients and ensure that they make ethically sound decisions around service delivery in a pandemic.

Future research can focus on a mixed method, starting with interviews with nurse educators about how they teach students to manage the family of a relative receiving end-of-life care or who has just died. The interviews can be followed by a questionnaire to measure to which extent junior nursing students know about providing the family of a relative with end-of-life care.

## Conclusion

The broader study indicated participants’ experiences of a sudden disruption to their family structure, leading to a measure of adjustment, which was hard to deal with. The study highlighted that the community of nurses should assist family members in traumatic circumstances by showing compassion in various ways. Trusting relationships during the pandemic were essential, and the need for nurses to show respect for human lives was at the core during their interactions in sharing information with family members. The study contributes to the awareness of effective interaction of nurses while sharing information with family members, as during COVID-19, it was an opportunity to assess the essential needs of families, such as providing support in relieving their emotional pain. Families have a longing for nearness to their relatives who are seriously ill; that is more than only the physical proximity to them. The underlying feelings of hurt that family members experienced came to the fore during the individual interviews and emphasised the need for family members of patients to be comforted. It was found that ‘the waves of hurt’ of family members (sometimes feeling better and sometimes worse) that were created by the unforeseen impact of COVID-19 was a vulnerable situation of unpredictability and fear of losing a loved one. The importance of the nurse as the close and trusting support for hurting families was confirmed in the findings.
